# Multifaceted Genes in Amyotrophic Lateral Sclerosis-Frontotemporal Dementia

**DOI:** 10.3389/fnins.2020.00684

**Published:** 2020-07-07

**Authors:** Ramya Ranganathan, Shaila Haque, Kayesha Coley, Stephanie Shepheard, Johnathan Cooper-Knock, Janine Kirby

**Affiliations:** ^1^Sheffield Institute for Translational Neuroscience (SITraN), The University of Sheffield, Sheffield, United Kingdom; ^2^Department of Biochemistry and Biotechnology, University of Barishal, Barishal, Bangladesh

**Keywords:** ALS, FTD, C9orf72, RNA processing, autophagy, protein aggregation

## Abstract

Amyotrophic lateral sclerosis and frontotemporal dementia are two progressive, adult onset neurodegenerative diseases, caused by the cell death of motor neurons in the motor cortex and spinal cord and cortical neurons in the frontal and temporal lobes, respectively. Whilst these have previously appeared to be quite distinct disorders, in terms of areas affected and clinical symptoms, identification of cognitive dysfunction as a component of amyotrophic lateral sclerosis (ALS), with some patients presenting with both ALS and FTD, overlapping features of neuropathology and the ongoing discoveries that a significant proportion of the genes underlying the familial forms of the disease are the same, has led to ALS and FTD being described as a disease spectrum. Many of these genes encode proteins in common biological pathways including RNA processing, autophagy, ubiquitin proteasome system, unfolded protein response and intracellular trafficking. This article provides an overview of the ALS-FTD genes before summarizing other known ALS and FTD causing genes where mutations have been found primarily in patients of one disease and rarely in the other. In discussing these genes, the review highlights the similarity of biological pathways in which the encoded proteins function and the interactions that occur between these proteins, whilst recognizing the distinctions of *MAPT*-related FTD and *SOD1*-related ALS. However, mutations in all of these genes result in similar pathology including protein aggregation and neuroinflammation, highlighting that multiple different mechanisms lead to common downstream effects and neuronal loss. Next generation sequencing has had a significant impact on the identification of genes associated with both diseases, and has also highlighted the widening clinical phenotypes associated with variants in these ALS and FTD genes. It is hoped that the large sequencing initiatives currently underway in ALS and FTD will begin to uncover why different diseases are associated with mutations within a single gene, especially as a personalized medicine approach to therapy, based on a patient’s genetics, approaches the clinic.

## Introduction

Amyotrophic lateral sclerosis (ALS) is an adult onset neurodegenerative disorder caused by progressive loss of upper motor neurons in the motor cortex and brainstem and lower motor neurons in the spinal cord (Hardiman et al., 2017). It has an incidence of 2–3 per 100,000 and a lifetime risk of 1 per 400 individuals (Brown and Al-Chalabi, 2017). Disease onset occurs most frequently in the limbs, characterized by a loss of dexterity in the fingers or a mild limp, whilst bulbar onset, which occurs in 20–25% of cases, is characterized by a slurring of speech (dysarthria) or difficulty swallowing (dysphagia). Less than 3% of cases are due to respiratory onset, with shortness of breath (dyspnea) being the most common symptom (Gautier et al., 2010). As the disease rapidly progresses, muscle wasting, fasciculations and weight loss occur, with death usually due to respiratory failure 32 months following symptom onset (Cooper-Knock et al., 2013).

Amyotrophic lateral sclerosis clinical features may also be accompanied by cognitive impairment in up to 50% of patients, whilst up to 15% may develop symptoms which are clinically diagnosed as frontotemporal dementia (FTD), resulting in a clinical diagnosis of ALS-FTD (Nguyen et al., 2018). FTD is the second most common form of presenile dementia after Alzheimer’s disease, accounting for 3–26% of cases of dementia in individuals under 65 years of age, depending on population (Bang et al., 2015). Whilst FTD is a clinical diagnosis, evidence of degeneration of the neurons in the frontal and temporal lobes upon post-mortem allows the pathological diagnosis of frontotemporal lobar degeneration (FTLD) (Mackenzie and Neumann, 2016). FTD can be divided into three different clinical subtypes. Behavioral variant FTD (bvFTD) is characterized by personality changes such as disinhibited behavior, apathy and loss of empathy. In contrast, semantic variant primary progressive aphasia (svPPA or svFTD) is characterized by individuals having difficulties understanding the meaning of words or naming objects or people, whereas non-fluent variant PPA (nfvPPA or nfvFTD) is when individuals have difficulties in pronunciation, grammar and fluency of speech (Bang et al., 2015).

Whilst the majority of ALS and FTD cases are sporadic (sALS and sFTD), with no family history of disease, in around 10% of cases ALS is inherited, usually in an autosomal dominant manner with an adult onset (fALS) (Brenner and Weishaupt, 2019). In FTD, it is estimated that 10–30% shows autosomal dominant inheritance (fFTD), though this figure may be increased to approximately 40% when a history of neurodegenerative disease is included (Sirkis et al., 2019). To date, 5 ALS-FTD genes have been recognized along with 24 ALS-associated genes and 3 FTD-associated genes (Table 1). This review will firstly summarize the genes that have been recognized as ALS-FTD genes (defined as FTDALS loci on Online Mendelian Inheritance in Man^1^) before summarizing other known ALS and FTD causing genes where mutations have been found primarily in patients of one disease and rarely in the other. Finally, a brief comment on the other ALS and FTD genes allocated an ALS or FTD loci number is provided for completeness and to highlight the similarity of biological pathways that are implicated in both disorders, supporting the proposal that these two disorders represent either end of a disease spectrum. Whilst some of these genes result in Mendelian inheritance of ALS, others act as risk factors. However, understanding why a particular mutation in a gene leads to ALS, FTD or both, currently remains unknown.

**TABLE 1:** Overview of known ALS-FTD, ALS, and FTD loci.

ALS Loci number	Chromosomal location	Gene	Onset	Inheritance	Implicated pathogenic mechanisms	Original References
**ALS-FTD genes**						
FTDALS1	9p21.2	*C9orf72*	Adult	AD	RNA processing; nucleocytoplasmic transport defects; proteasome impairment; autophagy; inflammation; protein aggregation (DPRs)	DeJesus-Hernandez et al., 2011; Renton et al., 2011
FTDALS2	22q11.23	*CHCHD10*	Adult	AD	Mitochondrial function, synaptic dysfunction	Bannwarth et al., 2014
FTDALS3	5q35.3	*SQSTM1*	Adult	AD	Proteasome impairment; autophagy; protein aggregation; axonal defects; oxidative stress	Fecto et al., 2011
FTDALS4	12q14.2	*TBK1*	Adult	AD	Autophagy; inflammation; mitochondrial dysfunction	Cirulli et al., 2015; Freischmidt et al., 2015
FTDALS5	16p13.3	*CCNF*	Adult	AD	Autophagy, axonal defects, protein aggregation	Williams et al., 2016
**Predominantly ALS genes also found with FTD**						
ALS6	16p11.2	*FUS*	Adult	AD (AR)	RNA processing; nucleocytoplasmic transport defects; stress granule function; protein aggregation	Kwiatkowski et al., 2009; Vance et al., 2009
ALS10	1p36.22	*TARDBP*	Adult	AD	RNA processing; nucleocytoplasmic transport defects; stress granule function; protein aggregation	Sreedharan et al., 2008
ALS12	10p13	*OPTN*	Adult	AD (AR)	Autophagy; protein aggregation; inflammation	Maruyama et al., 2010
ALS15	Xp11.21	*UBQLN2*	Adult	X-LD	Proteasome impairment; autophagy; protein aggregation; oxidative stress; axonal defects	Deng et al., 2011
ALS22	2q35	*TUBA4A*	Adult	AD	Cytoskeleton	Smith et al., 2014
ALS13	12q24.12	*ATXN2*	Adult	AD	RNA processing	Elden et al., 2010
**Predominantly FTD genes also found with ALS**						
ALS14	9p13.3	*VCP*	Adult	AD	Autophagy; proteasome impairment; defects in stress granules; protein aggregation; mitochondrial dysfunction	Johnson et al., 2010
ALS17	3p11.2	*CHMP2B*	Adult	AD	Autophagy; protein aggregation	Parkinson et al., 2006
**Known ALS genes**						
ALS1	21q22.11	*SOD1*	Adult	AD (AR)	Oxidative stress; protein aggregation; mitochondrial dysfunction, axonal defects, proteasome impairment apoptosis	Rosen et al., 1993
ALS2	2q33.1	*ALS2*	Juvenile	AR	Intracellular trafficking	Hadano et al., 2001; Yang et al., 2001
ALS4	9q34.13	*SETX*	Juvenile	AD	RNA processing	Chen et al., 2004
ALS5	15q21.1	*SPG11*	Juvenile	AR	Axonal defects	Orlacchio et al., 2010
ALS8	20q13. 32	*VAPB*	Adult	AD	Proteasome impairment; intracellular trafficking	Nishimura et al., 2004
ALS9	14q11.2	*ANG*	Adult	AD	RNA processing	Greenway et al., 2006
ALS11	6q21	*FIG4*	Adult	AD	Intracellular trafficking	Chow et al., 2009
ALS16	9p13.3	*SIGMAR1*	Juvenile	AD and AR	Proteasome impairment; intracellular trafficking	Luty et al., 2010; AL-Saif et al., 2011
ALS18	17p13.2	*PFN1*	Adult	AD	Axonal defects	Wu et al., 2012
ALS19	2q34	*ERBB4*	Adult	AD	Neuronal development	Takahashi et al., 2013
ALS20	12q13.13	*hnRNPA1*	Adult	AD	RNA processing	Kim et al., 2013
ALS21	5q31.2	*MATR3*	Adult	AD	RNA processing	Johnson et al., 2014
ALS23	10q22.2	*ANXA11*	Adult	AD	Intracellular trafficking	Smith et al., 2014
ALS24	4q33	*NEK1*	Adult	AD	Intracellular trafficking	Brenner et al., 2016
ALS25	12q13.3	*KIF5A*	Adult	AD	Axonal defects; intracellular trafficking	Nicolas et al., 2018
ALS	3p21.1	*GLT8D1*	Adult	AD	Ganglioside synthesis	Cooper-Knock et al., 2019
**Known FTD genes**						
FTD	17q21.2	*MAPT*	Adult	AD	Axonal defects, protein aggregation	Hutton et al., 1998
FTD	17q21.31	*GRN*	Adult	AD	Baker et al., 2006; Cruts et al., 2006	Inflammation; protein aggregation
FTD	6q27	*TBP*	Adult	AD	RNA processing	Olszewska et al., 2019

*AD, autosomal dominant; AR, autosomal recessive; X-LD, X-linked inheritance.*

## ALS-FTD Genes

Mutations in five genes have been recognized as being implicated in ALS-FTD families. These are the GGGGCC (G4C2) hexanucleotide repeat expansion (HRE) in *C9orf72* (FTDALS1), and missense and/or loss of function mutations in *CHCHD10* (FTDALS2), *SQSTM1* (FTDALS3), and *TBK1* (FTDALS4). In addition, *CCNF* has also been reported as an ALS-FTD gene and is referred in this review as FTDALS5. All of these genes encode proteins with a function in autophagy, except CHCHD10, which localizes to the mitochondria.

### FTDALS1: Chromosome 9 Open Reading Frame 72 (*C9orf72*)

**FIGURE 1:**
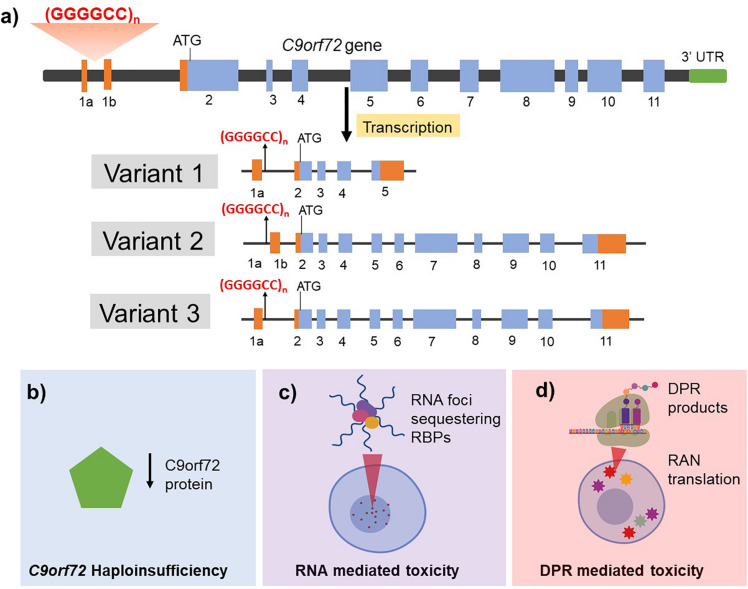
Structure of the *C9orf72* gene **(a)** and the proposed mechanisms of *C9orf72* related toxicity in driving ALS/FTD pathogenesis **(b–d)**. **(a)** *C9orf72* has 11 exons and is transcribed into three different mRNA transcripts: variant 1 results in translation of the short protein isoform while variants 2 and 3 both generate the long protein isoform. The non-coding introns are represented in orange and the coding exons are shown in blue. The hexanucleotide repeat expansion is present within intron 1 region and is retained in the pre-mRNA transcript in variants 1 and 3 whereas it is present in the promoter region in variant 2. The G4C2 HRE is proposed to cause neurotoxicity by three mutually compatible mechanisms. **(b)** Haploinsufficiency of endogenous C9orf2 protein by incorporation of the repeat in the transcript leading to reduced production and function of normal C9orf72 protein. **(c)** RNA mediated toxicity by the formation of RNA foci that sequester RNA binding proteins (RBPs) and **(d)** DPR mediated toxicity resulting from repeat associated non-AUG (RAN) translation of mRNA transcripts retaining hexanucleotide repeats thus generating five different dipeptide repeat (DPR) products.

Linkage and genome wide association studies in several families presenting with ALS/FTD, ALS or FTD revealed the diseases to segregate with a locus on chromosome 9p21 (Morita et al., 2006; Vance et al., 2006; Laaksovirta et al., 2010; Shatunov et al., 2010). In 2011, the pathogenic G4C2 hexanucleotide repeat expansion (HRE) in intron 1 of chromosome 9 open reading frame 72 (*C9orf72*) (Figure 1) was found to be the most common cause of familial ALS and FTD (DeJesus-Hernandez et al., 2011; Renton et al., 2011) with ∼40% fALS and 25% fFTD carrying the *C9orf72* repeat expansion (Majounie et al., 2012). However, the frequency of *C9orf72*-related ALS-FTD (and *C9orf72*-ALS and *C9orf72*-FTD) patients positive for the presence of repeat expansion in *C9orf72* varies between different populations and ethnicities; whilst the *C9orf72* G4C2 HRE is the most frequent cause of ALS-FTD, ALS and FTD in European and North American populations, it was found to be extremely rare in Asia and the Middle East (Majounie et al., 2012). Whilst it is currently unclear why some patients with the *C9orf72* expansion develop ALS or FTD while others manifest a combination of both, the discovery of *C9orf72* in causing both ALS and FTD has strengthened the genetic link between these neurodegenerative disorders. *C9orf72*-related ALS-FTD has an autosomal dominant mode of inheritance with evidence of incomplete penetrance (Majounie et al., 2012). Anticipation has been reported in some families (Gijselinck et al., 2016; Van Mossevelde et al., 2017b), however, this phenomenon has been found to be inconsistent, with other studies having reported no association between age of onset and repeat length (Dols-Icardo et al., 2014) or even an inverse correlation between expansion size and age of onset in successive generations, with contractions in the repeat size also reported in subsequent generations (Fournier et al., 2019; Jackson et al., 2020). Some of these contradictory findings may be attributed to the age at collection of sample, owing to the dynamic nature of repeat size relative to age, the methodology used and the source of samples being compared (peripheral blood versus brain autopsy tissue) (van Blitterswijk et al., 2013a; Fournier et al., 2019).

Although, the exact cut-off for repeat size that would result in pathogenicity has not been clearly defined, neurologically healthy non-carriers typically present with less than 20 repeats, while repeat expansion mutation carriers show more than 30 repeats on repeat primed PCR (Renton et al., 2011). Southern blotting of the region has estimated that repeat lengths of several hundred to several thousand are associated with *C9orf72*-related ALS-FTD, though smaller repeat sizes have been found to co-segregate with disease (Van Mossevelde et al., 2017a). Conventional techniques such as short-read next generation sequencing (NGS) limit accurate repeat sizing of the larger repeats, and somatic instability has been reported, thereby contributing to the variability in repeat size in different tissues from the same individual, such as in comparisons between blood and central nervous system (CNS) tissue (Vatsavayai et al., 2019). Studies have described an inverse relationship between repeat expansion size and disease duration with *C9orf72*-related ALS-FTD presenting an earlier age of symptom onset, a higher incidence of bulbar onset and shorter disease duration (Majounie et al., 2012; van Blitterswijk et al., 2013b; Suh et al., 2015; Trojsi et al., 2019). However, other studies have also reported disease durations between 1 and 22 years (Majounie et al., 2012; Woollacott and Mead, 2014). Sex has been reported to be a risk factor in driving phenotype with one study showing females have a higher prevalence for *C9orf72* HRE in ALS in a meta-analysis study (Trojsi et al., 2019) whilst males presented with a shorter survival time based on Cox proportional hazard regression multivariate analysis (Curtis et al., 2017). It is noteworthy that *C9orf72* repeat expansions have been shown to be associated with a number of neurodegenerative conditions including Parkinson disease, Alzheimer’s disease, Huntington-disease like syndrome among others (Woollacott and Mead, 2014; Cooper-Knock et al., 2015c).

The function of endogenous C9orf72 protein is not fully characterized although it has been identified as a guanine exchange factor (GEF), with both Rho and Rab-GTPase GEF activity (Iyer et al., 2018). It has also been shown that the C9orf72 protein interacts with the Rab1a and Unc-51-like kinase 1 (ULK1) autophagy initiation complex, with the C9orf72 protein regulating the trafficking of the ULK1 complex to the phagophore (Webster et al., 2016). As such, a reduction in C9orf72 protein would lead to reduced autophagy and accumulation of p62-positive aggregates, similar to those seen upon neuropathological examination of patients.

Whilst the exact mechanism of action of how the HRE in *C9orf72* causes neurodegeneration remains to be fully elucidated, three mutually compatible mechanisms have been proposed including haploinsufficiency of endogenous C9orf72 protein, loss of function and/or toxic gain of function following the formation of RNA foci and toxic gain of function of the dipeptide repeat (DPR) protein inclusions (Figure 1).

#### Haploinsufficiency of C9orf72

The *C9orf72* gene contains 12 exons (1a, 1b, 2–11) and has three well characterized transcripts which produce two protein isoforms (Figure 1) (Balendra and Isaacs, 2018) though additional alternatively spliced and protein coding transcripts have been identified (see Ensembl ENSG00000147894). The HRE is located in the pre-mRNA transcript of variant 1 and 3 but in the promoter region of variant 2. It is noteworthy that variants 1 and 3 are predominantly expressed in the brain. Additionally, HRE dependent epigenetic changes such as hypermethylation of the *C9orf72* gene locus has been reported and associated with disease duration and advanced age of onset (Nordin et al., 2015; Gijselinck et al., 2016; Zhang M. et al., 2017). Studies have shown that transcript sequences upstream of the repeat are increased relative to those downstream which might imply that the transcription was aborted due to the presence of repeat. Consequently, reduced levels of the transcript variants have been reported in blood lymphocytes (Ciura et al., 2013), induced pluripotent stem cells (iPSCs)-derived neurons (Shi et al., 2018) and post mortem tissue of *C9orf72*-related ALS and FTD patients (van Blitterswijk et al., 2013b). *C9orf72* knockdown in zebrafish was shown to produce defective axon generation and motor deficits (Ciura et al., 2013) indicating that C9orf72 protein might play a role in neuronal health. In contrast, specific knockdown of *C9orf72* in mouse brain by antisense oligonucleotides (Lagier-Tourenne et al., 2013) or full ablation of *C9orf72* (*C9orf72*^–/^*^–^*) in neuron-specific (Atanasio et al., 2016) or all tissues (Sudria-Lopez et al., 2016) in mice models showed no neurodegenerative phenotype, although ablated mice developed an autoimmune phenotype and showed reduced survival. This suggests that haploinsufficiency alone may not be sufficient to cause disease and a combination of aberrant pathways such as gain of toxic function with loss of endogenous C9orf72 protein function might therefore be required for disease pathogenesis.

#### RNA Foci Formation

The hexanucleotide repeat DNA sequences are bidirectionally transcribed resulting in the production of G4C2 sense and C2G4 antisense transcripts retaining the repeat expansions. Hexanucleotide repeat-retaining RNA forms secondary structures (such as a G-quadruplex) in which the abnormal RNA accumulate to form RNA foci. These RNA foci have been shown to be present in post-mortem brain and spinal cord tissues of *C9orf72*-related ALS-FTD patients whilst being absent in age-matched non-ALS/FTD neurologically healthy controls (Zu et al., 2013). Sense and antisense RNA transcripts get transported to the cytoplasm and have been detected in the cytoplasm of patient post mortem tissue (Mizielinska et al., 2013; Cooper-Knock et al., 2015b) and RNA foci have been found in many cell lines and patient biosamples, such as leukocytes, fibroblasts, and iPSC-derived motor neurons (Gendron and Petrucelli, 2018). The aggregation of RNA foci are dynamic and result from association and dissociation of RNA binding proteins (RBPs) which results in loss of their function. Research has shown that antisense foci were observed to be higher in the Purkinje neurons in cerebellum and motor neurons whereas sense foci were significantly increased in the granule neurons in the cerebellum obtained post mortem from *C9orf72*-related ALS or *C9orf72*-related ALS-FTD patients, as detected by fluorescence *in situ* hybridization (Cooper-Knock et al., 2014, 2015b) and RT-PCR (Zu et al., 2013).

Several studies have aimed to identify the RBPs that are sequestered within the C9orf72 RNA foci and have found hnRNPA1/3, PUR-a, ADARB2, Nucleolin, SRSF2, and ALYREF in post mortem CNS tissue, iPSCs-derived cortical neurons reprogrammed from C9orf72-related ALS-FTD patient fibroblasts, neuronal cell lines or a *Drosophila* model of *C9orf72* ALS (Donnelly et al., 2013; Sareen et al., 2013; Cooper-Knock et al., 2014; Hautbergue et al., 2017). Gene ontology and transcriptomic analyses have revealed that the formation of foci result in transcriptional profiles unique to *C9orf72*-related ALS and FTD when compared to other genetic causes of ALS and FTD. Changes in gene expression are associated with various cellular pathways including unfolded protein response (UPR), RNA splicing, inflammatory response, cell signaling and synaptic transmission (Cooper-Knock et al., 2015a; Prudencio et al., 2015). Taken together, these studies provide evidence for both a loss of function of the RNA binding proteins and a potential gain of toxic function of the downstream effects of the RNA foci formation in driving *C9orf72*-related ALS-FTD pathogenesis.

#### Toxicity by DPR

The *C9orf72* HRE sense and antisense RNA transcripts can get translated by a non-canonical mechanism of repeat associated non-ATG (RAN) translation resulting in the production of dipeptide repeats. RAN translation occurs in the absence of an AUG start codon resulting in multiple reading frames of a single repeat transcript. These DPRs can be generated from the sense and/or antisense transcripts resulting in the formation of five different products: poly-GA and poly-GR are translated from the sense GGGGCC strand whereas poly-PA and poly-PR are translated from the antisense CCGGGG strand; poly-GP is translated from both strands. DPRs have been shown to aggregate in the cytoplasm and appear as star-shaped inclusions in both neurons and glia (Mann et al., 2013; Schludi et al., 2015). Sense-derived poly-GA aggregated seem to be more frequent than others, however, both sense and antisense DPRs have been observed in neocortex, hippocampus, and thalamus (Cooper-Knock et al., 2012; Schludi et al., 2015). poly-GA and poly-PR inclusions were found to be more frequent in the granular layer of cerebellum and CA3/4 regions, respectively, in C9orf72-FTD compared to *C9orf72*-ALS and *C9orf72*-related ALS-FTD patients (Schludi et al., 2015). In contrast, DPRs were rarely observed in the brainstem and spinal cord. Arginine rich DPRs (poly-PR and poly-GR) have been documented to be toxic and contributory to neurodegeneration although these studies were performed on primary neuronal cultures and animal models (May et al., 2014; Mizielinska et al., 2014; Moens et al., 2017). DPR inclusions stain positive for p62 (SQSTM1), which is a component of ubiquitin-proteasome system (and which is also mutated in ALS-FTD) whilst TDP-43 pathology is found to be variable, with TDP-43 inclusions not always present in the same neurons as DPR inclusions (Cooper-Knock et al., 2012, 2015a; Mann et al., 2013; Schludi et al., 2015; Hautbergue et al., 2017).

In a *Drosophila* model of *C9orf72*-related ALS and FTD, it was demonstrated that neurodegeneration was mediated through DPRs rather than RNA foci (Mizielinska et al., 2014; Tran et al., 2015). Firstly, only pure repeats rather than stop-codon interrupted “RNA only” repeats led to a neurodegenerative-like phenotype in the flies (Mizielinska et al., 2014). A second study generated several uninterrupted repeat constructs including 5, 12, 40, and 160 G4C2 repeats with flanking intron and exon sequences (Tran et al., 2015). Although the fly expressing this 160 G4C2 repeat formed abundant RNA foci in neurons and glia, no DPR were produced and it did not develop neurotoxicity (Tran et al., 2015). In contrast, BAC transgenic mice models of *C9orf72*-related ALS-FTD showed RNA foci and DPR inclusions without development of a neurodegenerative-like phenotype or behavioral abnormalities (O’Rourke et al., 2015; Peters et al., 2015). Recently, a DPR-only mouse expressing poly(GA)_149_ conjugated to cyan fluorescent protein developed p62-positive poly-GA inclusions in motor neurons and interneurons of spinal cord, brain stem and in cerebellar nuclei, with motor deficits occurring at 4 months. However, there was no impairment to learning and memory (Schludi et al., 2017).

Expansions of G4C2 which are translated to form DPRs have been shown in *Drosophila* models and *C9orf72*-ALS iPSC derived neurons to disrupt nucleocytoplasmic transport (NCT), both export of nuclear RNA and import of nuclear proteins, through binding to many of the nuclear pore complex proteins (Freibaum et al., 2015). RanGAP, a key regulator of nucleocytoplasmic transport, was found to bind to both the G4C2 RNA and the DPR protein poly(GA) thereby causing defective nucleocytoplasmic transport in *Drosophila* and mouse models of *C9orf72*-ALS and iPSC derived neurons (Zhang et al., 2015, 2016). Poly(PR) and poly(GR) were also found to interact with RNA binding proteins and other low complexity domain proteins, including those in the nuclear pore complex (Lee et al., 2016) and using *Xenopus laevis* oocytes, poly(PR) was shown to bind and block the central channel of the pore (Shi et al., 2017). However, in the SHSY-5Y neuronal cell line and iPSC-derived neurons, it was shown that poly(GR) and poly(PR) had no effect on active nucleocytoplasmic transport, though poly(GA) deficits were observed (Vanneste et al., 2019). Thus, there are likely to be specific pathogenic mechanisms associated with the different DPRs.

In summary, many studies have been conducted to elucidate the pathogenesis of the *C9orf72* HRE in causing neurodegeneration and cognitive dysfunction without obtaining clear conclusions. Several factors including background of the animal studies, overexpression of mutation and experimental design can contribute to the variable results. It remains to be determined whether loss of C9orf72 protein function and toxic gain of function by RNA foci formation and DPR inclusion act in a concerted manner to manifest neurodegeneration in ALS and patient-derived cell models may be a more appropriate model for addressing these questions as they carry the HRE in a natural genetic context and the protein is expressed at physiological levels. However, a recent paper described that knocking down one or both endogenous *C9orf72* alleles in transgenic mice expressing either 66 repeats or 450 repeats led to reduced autophagy and enhanced DPR accumulations, cognitive deficits, hippocampal neuron loss and glial activation (Zhu et al., 2020). Thus, this work demonstrated that there is a synergy between the loss of C9orf72 protein and the toxic gain of function mechanisms. In addition, these mechanisms offer targets for novel therapeutics and the antisense oligomer strategy currently in clinical trials may offer a disease-modifying therapy.

### FTDALS2: Coiled-Coil Helix Coiled-Coil Helix Domain Containing Protein 10 (CHCHD10)

*CHCHD10* was initially associated with ALS when it was shown to segregate with disease in a family presenting with a complex phenotype including ALS, FTD, cerebellar ataxia, and myopathy (Bannwarth et al., 2014). Subsequently, several cohorts of ALS and ALS-FTD patients were screened for *CHCHD10* mutations and a number of candidate rare, predicted deleterious mutations were identified (Johnson et al., 2014; Dols-Icardo et al., 2015). However, it has been shown more recently that many of the proposed mutations are present at similar frequency in controls (Marroquin et al., 2016), perhaps because exome sequencing studies typically give poor coverage of the *CHCHD10* gene which leads to a propensity for false positives. Whole genome sequencing, where coverage of the *CHCHD10* is complete, revealed that there was no significant burden of disease-associated mutations in sporadic ALS patients (Project MinE ALSSC, 2018). In fact, with increased coverage of control cohorts most of the ALS-associated mutations have been found at comparable frequency in controls despite *in vitro* and *in vivo* evidence for toxicity. Of the remaining mutations there is a notable association with complex phenotypes including motor neuron degeneration but not typical ALS. The exception is c.44G > T (p.Arg15Leu) which has been identified in both sporadic and familial ALS cases with variable penetrance but is virtually absent in control databases (Project MinE ALSSC, 2018). Some of these cases have additional phenotypes such as hearing loss which may represent a distinct pathogenic process. However, assessment of TDP-43 pathology in these cases, which is arguably the molecular hallmark of ALS, is not yet available. CHCHD10 is localized to the mitochondria and patients with mutations in *CHCHD10* demonstrated abnormal mitochondrial morphology and respiratory chain deficiencies (Bannwarth et al., 2014).

### FTDALS3: Sequestosome 1 (*SQSTM1*):

SQSTM1, also known as p62, is a ubiquitin binding protein that is present in a variety of ubiquitinated inclusions associated neurodegenerative diseases including ALS and FTD. Mutations in this gene were originally associated with Paget’s disease of bone (PDB), a progressive skeletal disorder associated with an increased bone turnover producing localized lesions and bone pain (Rea et al., 2014). Missense mutations in the ubiquitin associated domain (UBA) or truncation mutations that cause partial or complete remove of the UBA account for 25–50% of familial PDB and 5–10% of sporadic PDB. However, the mutations in *SQSTM1* associated with ALS, FTD, and ALS-FTD cases are found throughout the gene, including the UBA, thereby impacting on many of the other pathways that the SQSTM1 protein participates in Rea et al. (2014). Functional domains include a light chain 3 (LC3) interacting region (LIR) that interacts with LC3 to promote autophagy, a KEAP1 interacting region (KIR) which binds KEAP1 competitively with NRF2 to regulate oxidative stress response and the PB1 domain, that interacts with several proteins which impact on neuronal survival and inflammation (Ma et al., 2019). Whilst *SQSTM1* mutations have been found in individuals who also carry a *C9orf72* expansion (Almeida et al., 2016; Kovacs et al., 2016), the pathogenicity of *SQSTM1* mutations has been demonstrated in zebrafish, which showed behavioral abnormalities as well as disrupted autophagy and shorter axons following knockdown of the *SQSTM1* ortholog. Importantly, these features were rescued by human SQSTM1 protein but not by the p.P392L common SQSTM1 mutation (Lattante et al., 2015). In addition, the KEAP1-NRF2 signaling pathway and oxidative response has been shown to be disrupted by *SQSTM1* mutations (Deng et al., 2019; Foster et al., 2019), a pathway originally found to be dysregulated in SOD1-ALS (Kirby et al., 2005), which was subsequently implicated in sALS (Sarlette et al., 2008). Finally, it has recently been shown that SQSTM1 co-localizes with misfolded MAPT and degrades the insoluble forms of the protein (Xu et al., 2019). In transgenic rTg4510 mutant MAPT mice, AAV-*SQSTM1* was administered to increase SQSTM1 protein expression and this resulted in reduced mutant insoluble MAPT and improved pathology, including reduced astrogliosis and microgliosis.

### FTDALS4: TANK-Binding Kinase (*TBK1*)

*TBK1* mutations were first linked to ALS (Cirulli et al., 2015; Freischmidt et al., 2015), FTD (Gijselinck et al., 2015; Le Ber et al., 2015) and ALS-FTD in 2015 (Pottier et al., 2015). Loss of function (LoF) mutations, including frameshifts, splice-site alterations, read-throughs and nonsense mutations have been reported to show definite or probable pathogenicity. The pathogenicity of missense mutations is less certain as some have also been found in controls (van der Zee et al., 2017) but such mutations in functional domains which impair target protein binding, or target or TBK1 phosphorylation, can also cause loss of function at the protein level (Freischmidt et al., 2015; Pozzi et al., 2017; van der Zee et al., 2017; de Majo et al., 2018). In addition, both LoF and missense mutations increase the risk of ALS/FTD (odds ratio 11.78 and 1.62, respectively) (Cui et al., 2018).

The mutation frequency of *TBK1* in ALS, FTD and ALS-FTD is reported to be from 0.4 to 1.7% (Gijselinck et al., 2015; van der Zee et al., 2017). More recently, a meta-analysis found LoF mutations in 1.0% and missense mutations in 1.8% of ALS and/or FTD, and suggests a higher prevalence in European populations compared to Asian populations (Cui et al., 2018). A separate paper also showed *TBK1* mutations to be the most important cause of ALS-FTD after *C9orf72* (Dols-Icardo et al., 2018).

*TBK1* codes the TBK1 (TANK-binding kinase 1) protein, a kinase which binds and phosphorylates proteins involved in innate immunity (Pilli et al., 2012), autophagy (Korac et al., 2013), and mitophagy (Heo et al., 2015). Protein targets include optineurin (*OPTN*) (ALS12) and p62 (*SQSTM1*) (FTDALS3), two ALS-FTD associated genes (Maruyama et al., 2010; Rea et al., 2014) and mutations in both of these genes have been found along with *TBK1* mutations in patients (Pottier et al., 2015; Borghero et al., 2016; Dols-Icardo et al., 2018; Lattante et al., 2019).

*TBK1* mutations have also been identified alongside the *C9orf72* expansion (Gijselinck et al., 2015; van der Zee et al., 2017), and mutations in *FUS* (Lattante et al., 2019), *TARDBP* (Freischmidt et al., 2015; de Majo et al., 2018), or *DCTN1* and *FUS* together (Muller et al., 2018). Interestingly, those harboring *TBK1* and *TARDBP* (de Majo et al., 2018) or *TBK1* and *FUS* (Freischmidt et al., 2015) mutations, showed earlier disease onset than those with *TBK1* alone (Freischmidt et al., 2015; Pozzi et al., 2017). One case showing *TBK1* and *C9orf72* mutations had a later disease onset; however, this was attributed to a shorter *C9orf72* expansion size of 59 repeats and variable penetrance of the *TBK1* mutation (Gijselinck et al., 2015). No further genotype-phenotype associations have been identified between *TBK1* mutation type or position, and clinical phenotype. Mutations occur throughout the *TBK1* gene, though missense variants cluster within the kinase and ubiquitin like domains (de Majo et al., 2018). *TBK1*-linked clinical phenotypes show variable age of onset, different rates of progression, and survival length (Gijselinck et al., 2015; Borghero et al., 2016; Pozzi et al., 2017; van der Zee et al., 2017; de Majo et al., 2018; Weinreich et al., 2019).

### Cyclin F (*CCNF*)

Mutation of *CCNF* was first identified using genome-wide linkage followed by exome sequencing within a large ALS-FTD pedigree (Williams et al., 2016). Subsequently additional variants in both ALS and FTD cases were identified accounting for 0.6–3.3% of fALS-FTD patients among different populations (Williams et al., 2016; Pan et al., 2017). Cyclin F, encoded by *CCNF*, is one of the components of an E3 ubiquitin-protein ligase complex also known as SCF^CyclinF^ (Skp1-Cul1-F-box E3 ubiquitin ligase complex) (Galper et al., 2017). Mutation of *CCNF* in neuronal cells causes errors in ubiquitination leading to ubiquitinated protein accumulation of SCF^CyclinF^ and TDP-43 as well as impairment of autophagosome-lysosome fusion (Williams et al., 2016; Lee et al., 2018). Recently, a *CCNF* mutation in a zebrafish model has been shown to have disrupted axonal outgrowth (Galper et al., 2017; Hogan et al., 2017). Further support for *CCNF* as an ALS-FTD gene comes from the finding that CCNF interacts with valosin containing protein (VCP) (ALS14), increasing VCP’s ATPase activity, which in turn promotes TDP-43 aggregation (Yu et al., 2019). Thus, for the purposes of this review, *CCNF* is described as FTDALS5.

## ALS Genes Subsequently Associated With ALS-FTD and FTD

There are many genes which have been characterized as causative for ALS where potentially pathogenic variants have also been described in FTD cases. Whilst this is rare, the co-occurrence of ALS and FTD being associated with mutations in these genes further strengthens the genetic linkage between these two disorders. These genes encode proteins associated with autophagy/ubiquitin proteasome system (UPS) or RNA processing. The exception is *TUBA4A*, which encodes a microtubule associated protein. In addition, intermediate CAG repeat expansions in *ATXN2* have been reported as a risk factor in ALS and ALS-FTD and a disease modifier in both ALS and FTD.

### ALS6: Fused in Sarcoma (*FUS*)

Fused in srcoma was initially identified as part of a fusion oncogene, *FUS-CHOP*, resulting from a *t*(12;16) (q13;p11) translocation event in malignant liposarcoma (Crozat et al., 1993; Rabbitts et al., 1993). Located at chromosome 16p11.2, *FUS* encodes a predominantly nuclear DNA/RNA binding protein which belongs to the FET protein family. As a functional component of the hnRNP complex, FUS is involved in many RNA processing activities, including transcription regulation, RNA transport and trafficking, pre-mRNA splicing, and miRNA processing. *FUS* consists of 15 exons which encode 526 amino acids. FUS has a multidomain structure consisting of an N-terminal glutamine-glycine-serine-tyrosine (QGSY) domain, three arginine-glycine-glycine rich domains (RGG1-3), an RRM, zinc finger motif (ZnF), and a highly conserved C-terminal NLS (Deng et al., 2014).

Mutations in *FUS* were first associated with autosomal recessive fALS, with additional screening revealing mutations in *FUS* to be causal in autosomal dominant ALS (Kwiatkowski et al., 2009; Vance et al., 2009). Further studies have shown that *FUS* mutations account for approximately 4% of fALS cases, and 1% of sALS cases. The vast majority of mutations are missense, with in-frame insertions and deletions occurring rarely. Although ALS-associated mutations occur throughout the whole length of the gene, most mutations are located in exons 3–6, encoding the N-terminal transcriptional activation domain, QGSY, and the nucleic acid binding domain RGG1, or in exons 12–15 which encode C-terminal nuclear binding domains RGG2 and RGG3, a ZnF domain and an NLS domain (Deng et al., 2014). Mutations within exons 12–15 have been found to be functional, whilst those in exons 3–6, which are also more commonly found in sALS, do not always segregate with disease. This indicates the presence of non-pathogenic variations, and incomplete penetrance, highlighting the complexity of the role of *FUS* in ALS pathogenesis. Screening of FTD patients subsequently identified several FUS mutations in patients with bv-FTD either with or without concurrent ALS though the frequency of FUS mutations is much rarer in FTD than ALS cases (Ticozzi et al., 2009; Blair et al., 2010; Van Langenhove et al., 2010; Huey, Ferrari et al., 2012).

Fused in srcoma plays an important role in RNA processing. Therefore, mutations in *FUS* have a negative impact on RNA transcription, alternative splicing, and mRNA transport and stabilization. It is evident that this results in widespread neuronal dysfunction, contributing to the ALS phenotype, although, how this occurs is not well understood. Several theories have been proposed, including gain- and loss-of-function mechanisms (Deng et al., 2014). Wild-type FUS is predominantly located in the nucleus, however, disease-causing mutations in the C-terminal NLS of *FUS*, including the most common *FUS* mutation, p.R521C, leads to FUS-positive neuronal cytoplasmic inclusions. The accumulation of FUS aggregates has been found in the neuronal cytoplasm and dendrites of ALS and FTLD patients. This disruption of nuclear import may result in toxic gain of cytoplasmic function and loss of nuclear function (Deng et al., 2014; Lopez-Erauskin et al., 2018). FUS inclusions have also been found in atypical FTLD cases (aFTLD-U) as one of the proteins in the ubiquitinated neuronal inclusions, as well as being found in glial cells (Neumann et al., 2009). None of these cases had mutations in the *FUS* gene.

Fused in srcoma was first found to have a role in RNA transcription when nuclear depletion of RNA polymerase II (RNAPII) resulted in an increase in cytoplasmic FUS (Zinszner et al., 1997). Further studies demonstrated the role of FUS in pre-mRNA splicing. FUS mediates the interaction between RNAPII and U1 snRNP, a splicing factor responsible for recognizing the 5′ splice junction (Yu and Reed, 2015). Beyond this, FUS has been identified as component of the spliceosome, and also interacts with other important splicing factors such as hnRNPA1 (Rappsilber et al., 2002; Zhou et al., 2013; Kamelgarn et al., 2016). Loss of FUS functionality affects the splicing of its target genes, contributing to widespread splicing dysfunction of genes involved in neuronal functions, such as *PPP2R2C* which is required for neurogenesis and *ACTL6B* which has a role in dendritic development (Reber et al., 2016). Beyond this, *FUS* mutations also result in the mislocalisation of U1 snRNP to the cytoplasm, and the aggregation of FUS, hnRNPA1, hnRNPA2 and SMN1 into stress granules (Takanashi and Yamaguchi, 2014; Yu et al., 2015).

Transcriptome analysis of human MNs generated from mutant FUS iPSCs, identified changes in expression levels of genes involved in cellular processes which have previously been associated with neurodegenerative disease, including cell adhesion. Also, TAF15 which is also a member of the FET family was found to be differentially expressed in FUS mutant MNs (De Santis et al., 2017). More recently, mutant FUS has been shown to affect important processes vital for neuronal functionality in mice. Studies using transgenic mice demonstrated that ALS/FTD-linked mutant FUS accumulates within axons, reducing intra-axonal translation which, in turn, causes early activation of the integrated stress response (ISR) and increased phosphorylation of eIF2α. Ultimately, this inhibits the protein synthesis of important RNAs, including those encoding ion channels and transporters essential for synaptic function (Lopez-Erauskin et al., 2018). Furthermore, suppressed protein synthesis and disrupted regulation of nonsense medicated decay was detected in fibroblast cells derived from FUS-related ALS cases (Kamelgarn et al., 2018).

### ALS10: TAR DNA Binding Protein (*TARDBP*)

*TARDBP* is located on chromosome 1p36.22 and encodes the transactive response DNA-binding protein 43 (TDP-43). Like FUS, TDP-43 is a predominantly nuclear DNA/RNA binding protein which is a member of the heterogeneous nuclear ribonucleoprotein (hnRNP) family (Sreedharan et al., 2008). The *TARDBP* gene consists of 6 exons, and has a similar structure to *FUS*; an N-terminal domain (NTD), 2 RNA recognition motifs (RRM1-2) which are involved in RNA and DNA binding, a nuclear localization signal and nuclear export signal, and a C-terminal glycine-rich domain (GRD) which is responsible for protein–protein interactions (Lagier-Tourenne et al., 2010; Baralle et al., 2013). TDP-43 was initially recognized as a transcription repressor protein which binds to the TAR regulatory element of human immunodeficiency virus-1 (HIV-1) (Ou et al., 1995). Further investigations have shown that TDP-43 has other important roles in RNA processing, including RNA transcription, pre-mRNA and pre-miRNA splicing, RNA transport and mRNA stability (Scotter et al., 2015).

Mutations in *TARDBP* are responsible for 4–5% of fALS cases and 1% of sALS cases, and are inherited in an autosomal dominant manner (Millecamps et al., 2010). *TARDBP* mutations cause an ALS phenotype consisting of classic ALS symptoms. Mutations in *TARDBP* have also been reported in patients with FTD, with and without ALS (Benajiba et al., 2009; Borroni et al., 2009; Kovacs et al., 2009; Pesiridis et al., 2009). The frequency of *TARDBP* mutations in patients with FTD is estimated at 1%, the majority presenting with bvFTD, though some patients do present with svFTD or nfvFTD at onset (Caroppo et al., 2016).

The majority of mutations are located in exon 6, which encodes the aggregation-prone C-terminal GRD. These mutations increase the aggregation potential of this protein. Ubiquitinated aggregates of TDP-43 are found in the cytoplasm of MNs of ALS and FTD patients, not just patients with *TARDBP* mutations (Neumann et al., 2006; Mackenzie and Rademakers, 2008; Johnson et al., 2009; Kirby et al., 2010). Given that 97% of fALS and sALS patients are positive for TDP-43 cytoplasmic inclusions, it is evident TDP-43 plays an important role in MN degeneration and disease pathogenesis (Sreedharan et al., 2008; Qin et al., 2014). In addition, TDP-43 positive inclusions are also found in 50% of FTLD cases (Neumann et al., 2006; Mackenzie et al., 2010). Although it is unknown how this occurs, it has been hypothesized that this may be due to toxic gain of cytoplasmic function and loss of nuclear function (Kabashi et al., 2010).

TDP-43 is functionally homologous to FUS and also has important functions in RNA metabolism. Mutations in *TARDBP* result in aberrant RNA processing on multiple levels; transcription regulation, alternative splicing and mRNA stability (Buratti and Baralle, 2008). Beyond regulating its own expression level by binding to the 3′ untranslated region (3′ UTR) of its mRNA, TDP-43 is also essential for maintaining normal expression levels and splicing patterns of over 1,000 mRNAs (Ayala et al., 2008; Polymenidou et al., 2011). Specifically, TDP-43 dysfunction results in the dysregulated expression of other ALS-associated proteins which also have roles in RNA metabolism, including FUS, ATXN2, and progranulin (PGRN) (Polymenidou et al., 2011; Sephton et al., 2011; Highley et al., 2014). Furthermore, dysfunction of TDP-43 results in defective alternative splicing of its target genes, including *hnRNAPA1*, which negatively impacts cellular stability (Butti and Patten, 2018). Additionally, TDP-43 is involved in the splicing of cryptic exons of particular mRNAs, such as *ATG4B* (autophagy related 4B cysteine peptidase). Splicing of cryptic exons produces aberrant mRNA products. These have been observed in the CNS of ALS and FTD patients and have been recently been linked to impaired autophagy (Ling et al., 2015; Torres et al., 2018). It is known that TDP-43 is a component of stress granules, but how this contributes to the ALS phenotype is unknown (Aulas and Vande Velde, 2015).

### ALS12: Optineurin (OPTN)

Amyotrophic lateral sclerosis-associated mutations in *OPTN*, which was previously implicated in glaucoma, were first identified in 2010 in six affected members of a Japanese pedigree with consanguineous marriages presenting with three different types of mutations: a homozygous deletion of exon 5, a homozygous nonsense p.Q398X mutation and a heterozygous missense p.E478G mutation (Maruyama et al., 2010). Subsequently, more than 20 mutations have been described although not all have been investigated in *in vitro* and *in vivo* disease models. The incidence of *OPTN* mutations in FTD is still under debate, as one study reported copy number variants in *OPTN* in 4.8% of FTD cases (Pottier et al., 2015) while another study, recruiting a larger cohort of 371 FTD cases, did not detect any mutations using whole exome sequencing (Rollinson et al., 2012). More recently, a patient with ALS-FTD was reported with compound heterozygous mutations, resulting in a 75–80% reduction in OPTN (Pottier et al., 2018).

OPTN is a highly conserved hexameric protein that is ubiquitously expressed with significantly high expression in skeletal muscles (Toth and Atkin, 2018). OPTN is known to interact with TBK1 (FTDALS4); in fact, a series of evolutionarily conserved serine residues precedes the hydrophobic core sequence in OPTN which bears homology to TBK1-binding site of TANK, another substrate of TBK1 (Wild et al., 2011). OPTN is involved in several cellular functions including autophagy, vesicular trafficking, Golgi maintenance [as evident from Golgi apparatus fragmentation in spinal motor neurons and glia in post mortem tissue obtained from an ALS-FTD patient (Kamada et al., 2014) and neuroinflammation (Toth and Atkin, 2018; McCauley and Baloh, 2019)]. OPTN has also been shown to regulate NFκB signaling wherein ALS associated mutations in *OPTN* showed increased immunoreactivity of microglia (McCauley and Baloh, 2019). OPTN-positive cytoplasmic inclusions in the CNS are not only seen in cases with *OPTN* mutations, but also in *C9orf72*, *FUS*, and *SOD1*-related cases (Bury et al., 2016). Interestingly, conditional loss of OPTN by Cre-loxP system in different cell types using a murine model (*Cnp-cre, Lyz2-cre, Gfap-cre*, and *Mnx1-cre* mice) showed RIPK1-mediated necroptosis resulting in axonal myelination pathology when OPTN was depleted in oligodendrocytes and myeloid cells, whereas no pathology was observed when OPTN expression was selectively removed in astrocytes and motor neurons (Ito et al., 2016), further confirming non-cell autonomous toxicity in driving neurodegeneration.

### ALS15: Ubiquilin 2 (*UBQLN2*)

Mutations in *UBQLN2*, which is localized on the X chromosome, were first identified in large ALS-FTD family in 2011 (Deng et al., 2011). Four mutations located within the proline-X-X (PXX) repeat region of the protein were subsequently found through additional screening of fALS cases with no male to male transmission. Further variants within or adjacent to the PXX repeat region have been identified in ALS, FTD, or ALS-FTD patients, though at rare frequencies (Williams et al., 2012; Gellera et al., 2013; Ugwu et al., 2015). As a member of the ubiquilin family, the protein is actively associated in the degradation of misfolded and redundant proteins through macroautophagy and the ubiquitin-proteasome system (Renaud et al., 2019). Mutations cause defective binding to the proteasome leading to interruption of the protein degradation, triggering mislocalisation of OPTN from Rab-11 positive endosomal vesicles as well as loss of binding of UBQLN2 to hnRNP proteins, including hnRNPA1 (ALS20) resulting in impaired RNA metabolism (Chang and Monteiro, 2015; Gilpin et al., 2015; Osaka et al., 2015). ALS-linked mutations in UBQLN2 gene were also found to be associated with dysfunction of autophagy, neuroinflammation, as well as the formation of stress granules, where mutations disrupted interaction with FUS (ALS6) and FUS-RNA complexes (Picher-Martel et al., 2015; Hjerpe et al., 2016; Alexander et al., 2018; Dao et al., 2018; Renaud et al., 2019).

### ALS22: Tubulin Alpha 4A (*TUBA4A*)

Mutations in Tubulin alpha 4A (*TUBA4A*) were identified as a very rare cause of ALS following the discovery of non-synonymous variants during whole exome sequencing of fALS index cases (Smith et al., 2014). Whilst patients had spinal-onset ALS, two cases developed FTD, whilst a third case had FTD in a first degree relative. Additional studies identified further cases of FTD and ALS with mutations in *TUBA4A*, though they were exceptionally rare, whilst other papers failed to find any *TUBA4A* mutations in ALS or FTD cohorts (Dols-Icardo et al., 2016; Perrone et al., 2017; Li et al., 2018). *TUBA4A* encodes an alpha tubulin subunit which combines with other alpha and beta tubulins to form microtubules. Mutant TUBA4A proteins showed altered incorporation into microtubules, thereby reducing the stability of the microtubule network in a dominant negative manner (Smith et al., 2014). Interestingly, a study identifying miR-1825 as decreased in both serum and plasma of sALS and fALS, was shown to directly target tubulin-folding co-factor b (TBCB) expression and this was associated with depolymerisation and degradation of TUBA4A protein in HEK293 cells (Helferich et al., 2018). Additional studies in zebrafish embryos expressing human TBCB displayed reduced levels of the TUBA4A zebrafish homolog and reduced axonal length and branching, whilst TBCB and TUBA4A proteins levels were inversely correlated in post-mortem brain cortex of fALS and sALS. Thus, *TUBA4A* is implicated not only through genetic mutations, but also by dysregulation of an upstream miRNA in both fALS and sALS cases.

### ALS13: Ataxin 2 (*ATXN2*)

A CAG repeat expansion encoding a polyglutamine repeat is found in ataxin 2 (*ATXN2*), a ubiquitously expressed protein involved in RNA processing, stress granule formation, endocytosis, calcium signaling and controlling metabolism and energy balance. In the normal population, the size varies between 13 and 31 CAG repeats, though 22–23 repeat are the most common (Velazquez-Perez et al., 2017). Repeats of over 35 are associated with fully penetrant spinocerebellar ataxia 2, with those 32–34 showing variable penetrance. Following identification that ATXN2 interacts with TDP-43, intermediate repeats of 27–33 were found to be a risk factor for ALS (Elden et al., 2010), with the intermediate CAG repeat interrupted with a CAA codon (Corrado et al., 2011). Subsequently, a meta-analysis of 9 studies highlighted that whilst there was an increased risk of ALS from 29 CAG/CAA repeats, significance was only reached for 31–33 repeats (Neuenschwander et al., 2014). Interestingly, repeat sizes of 27–28 were found to lower risk of ALS. More recently, a meta-analysis of 16 published studies, along with two large unpublished cohorts of ALS demonstrated an increased risk of ALS with 29–32 CAG/CAA repeats, and this risk increased with the number of repeats (Sproviero et al., 2017). This study also found 27 repeats to have a protective effect.

Following the link with ALS, the role of *ATXN2* intermediate repeats in FTD was investigated. Screening of ALS and FTD alongside other neurodegenerative diseases identified 30–33 repeats to be associated with ALS but not FTD (Ross et al., 2011). Subsequently, *ATXN2* CAG repeats of ≥29 were also found to be associated with ALS and familial ALS-FTD but not sporadic ALS-FTD or FTD (Lattante et al., 2014). A further study of 368 cases also found no significant correlation between FTD and *ATXN2* CAG repeat size though they did find that intermediate repeats (≥27) were associated with an earlier age at onset of FTD (Rubino et al., 2019).

Screening of ATXN2 has also identified expansions >34 in rare cases of both ALS (Corrado et al., 2011; Ross et al., 2011; Van Damme et al., 2011) and FTD (Baumer et al., 2014; Fournier et al., 2018) although no signs of ataxia were reported and neuropathological examination confirmed a diagnosis of ALS. As well as interacting with TDP-43, ATXN2 has also shown to bind to mutant FUS, with intermediate repeats binding both WT and mutant FUS proteins (Farg et al., 2013).

## FTD Genes Subsequently Associated With ALS-FTD and ALS

Several ALS genes identified through next generation sequencing have previously been identified as being associated with FTD or a syndrome incorporating FTD, such as inclusion body myopathy with Paget’s disease of bone and frontotemporal dementia (IBMPFD). These include *VCP* and *CHMP2B*.

### ALS14: Valosin Containing Protein (*VCP*)

Mutations in *VCP* have been described in ALS, FTD and inclusion body myopathy with Paget’s disease of bone and FTD (IBMPFD) which is an adult onset disorder characterized by muscle weakness, early onset PDB (see section “Cyclin F (*CCNF*)”) and FTD, though episodic memory is preserved (Kimonis, 1993). Mutations in VCP account for 1–2% of fALS cases, are found to be rare in sALS (Koppers et al., 2012) and whilst FTD is recognized in a third of IBMPFD patients, mutations have been found in FTD cases (Saracino et al., 2018; Wong et al., 2018).

Valosin containing protein (also called as CDC48 or p97) is a hexameric ATPase that is ubiquitously expressed and involved in diverse cellular functions including autophagy, endoplasmic reticulum (ER)- associated degradation (ERAD), chromatin remodeling, DNA repair and other protein quality control pathways (Wang et al., 2016; Shahheydari et al., 2017). ATPase has two domains, D1 and D2 and a regulatory N-domain. A majority of the mutations in *VCP* have been documented in the N-domain in patients with ALS and/or FTD (Abrahao et al., 2016; Wang et al., 2016; Shahheydari et al., 2017) although additional ALS and FTD mutations have been reported in the D1 domain (Wong et al., 2018). A study reported that mutations in the N-domain, an evolutionarily conserved region in VCP, results in poor hexamer assembly and reduced small ubiquitin-like modifier (SUMO)-ylation of VCP that diminishes its recruitment to stress granules and consequently affects ERAD in a *Drosophila* model of ALS/FTD. In contrast, a recent study that screened 48 patients with FTD reported identified 3 mutations that lie within the D1 domain of VCP and are hypothesized to affect ATPase binding activity (Wong et al., 2018). Interestingly, it has been reported that VCP interacts with FUS (ALS6) (Wang et al., 2015) and Cyclin F (CCNF) (proposed FTDALS5) (Yu et al., 2019) both of which are implicated in ALS. Mutations in *FUS*/*CCNF* were shown to increase ATPase activity of VCP in the cytoplasm, causing VCP to mislocalize to the cytoplasm (Yu et al., 2019) and trigger accumulation of polyubiquitinated proteins (Wang et al., 2015). VCP is also vital in mitochondrial quality control and IBMFTD patient fibroblasts carrying a mutation in VCP showed uncoupling of mitochondria, reduced mitochondrial membrane potential and ATP production (Bartolome et al., 2013), a feature that is also evident in *SIGMAR1* (ALS16) mutations.

### ALS17: Chromatin Modifying Protein 2B (*CHMP2B*)

Mutation of *CHMP2B* was initially identified in a large Danish family with FTD linked to chromosome 3 (termed FTD-3) (Skibinski et al., 2005). The splice site mutation c.532-1G > C results in the formation of two transcripts encoding two different proteins with a defective carboxyl terminus: CHMP2B^intron5^, where the intronic sequence between exons 5 and 6 is retained and a single valine is incorporated instead of the final 36 amino acids encoded by exon 6 and CHMP2B^del10^, where a cryptic splice site is used 10 bp from exon 6, resulting in the insertion of 29 novel amino acids. Subsequently, a Belgian family with FTD-3 was identified, where the c.493C > T mutation lead to truncation of the protein, with the loss of 49 amino acids (van der Zee et al., 2008). In contrast, mutations identified in *CHMP2B* that were associated with ALS were missense mutations (Parkinson et al., 2006).

CHMP2B is a component of the endosomal sorting complex required for transport III (ESCRT-III) complex, which is involved in the maturation of endosomes and autophagosomes. Using cellular and animal models, mutations in CHMP2B (both truncated and missense mutations) have been shown to disrupt endosomal-lysosomal trafficking, through accumulation and enlargement of endosomes (Cox et al., 2010; Zhang Y. et al., 2017; Vandal et al., 2018). The pathology of FTD-3 cases is distinguished by the presence of ubiquitin and p62 (*SQSTM1*; FTDALS3) positive inclusions, which are negative for TDP-43 and Tau (Holm et al., 2007).

## Other ALS and FTD Genes

**FIGURE 2:**
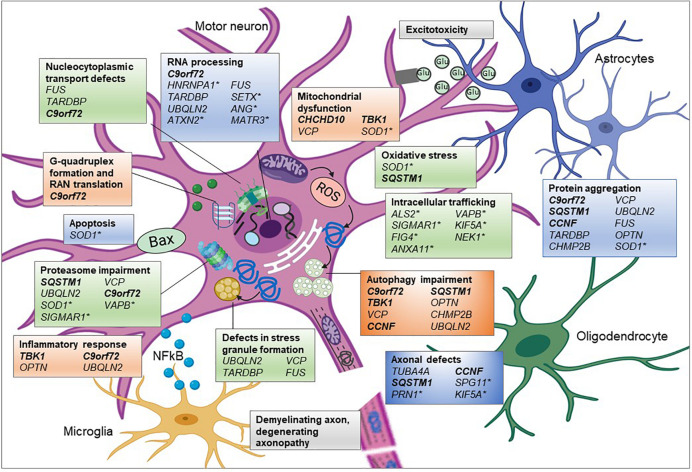
Pathogenic pathways associated with genetic variants of ALS. ALS is a complex disease affecting multiple interconnecting cellular pathways and dysfunction of these pathways has been associated with many of the genetic mutations. Proposed aberrant mechanisms include abnormal nucleocytoplasmic transport of RNA and RNA binding proteins (RBP) and altered RNA metabolism resulting from mislocalisation of RBPs. Mutations in RBP can undergo liquid-liquid phase separation thereby altering stress granule formation and propagating cytoplasmic protein aggregation. Overload of these misfolded proteins could burden the proteasome-ubiquitin system affecting timely clearance of abnormal proteins and downstream processes such as autophagy. Protein aggregation could influence microtubule dynamics resulting in abnormal anterograde and retrograde axonal transport of vesicular cargoes and mitochondria. See text for full information on how each of these genes associates with the mechanism shown. Glu, glutamate; genes in bold and italic, ALS-FTD genes;^∗^ = genes only associated with ALS and the remainder are associated with ALS and FTD in differing proportions. Created using Biorender.com.

In addition to those genes described above, there are other genes associated solely with fALS and fFTD, including “Pure” ALS genes such as SOD1 and “Pure” FTLD genes MAPT and Progranulin (Bennion Callister and Pickering-Brown, 2014). However, it is notable that many of these pure ALS genes also encode proteins that cluster into functional pathways associated with ALS-FTD genes, with mutations in the pure ALS gene affecting similar biological pathways. For example, numerous genes are associated with autophagy/proteasome impairment (*C9orf72, SQSTM1, TBK1, OPTN, VCP, UBQLN2*, and *CHMP2B*) and/or their proteins are found to be aggregated in cytoplasmic inclusions (C9orf72, SQSTM1, OPTN, VCP, UBQLN2, CCNF, FUS, TDP-43, and CHMP2B) (Figure 2). Many of these genes also encode proteins that have a role in RNA processing (*C9orf72*, *FUS, TARDBP*, and *UBQLN2*), whilst others are associated with and dysregulate the mitochondria (*CHCHD10; TBK1*; and *VCP*) or the cytoskeleton (*TUBA4A, CCNF*, and *SQSTM1*). SOD1-ALS, accounting for around 10% of fALS cases, is distinctive in that it is not associated with TDP-43 inclusions, unlike the majority of ALS cases. However, mutations in *SOD1* are associated with similar pathogenic mechanisms, such as disruption to protein quality control, mitochondrial dysfunction, dysregulated axonal transport and RNA processing, in addition to oxidative stress and excitotoxicity. Due to the wide range of biological pathways, gene silencing of SOD1 is currently in clinical trials as a therapeutic strategy for SOD1-ALS patients (van Zundert and Brown, 2017).

Many of the additional ALS genes can also be categorized into these pathways (Table 1 and Figure 2), such as *SETX, ANG, ATXN2, hnRNPA1*, and *MATR3*, which are all involved in RNA processing and *SPG11, KIF5A*, and *PFN1* that are associated with the cytoskeleton and mutations in which cause axonal defects (Alsultan et al., 2016). Many of the genes also encode proteins involved in trafficking components within the cell, such as endosomes (*ALS2, FIG4*, and *NEK1*) or in the unfolded protein response (*VAPB* and *SIGMAR1*). However, as research has investigated the effect of mutations within these genes, additional secondary pathways have been implicated, such as mutant SOD1 protein’s effect on protein homeostasis, gene expression and axonal transport, resulting in a complex interactome of direct and indirect effects, which ultimately lead to neurodegeneration.

**FIGURE 3:**
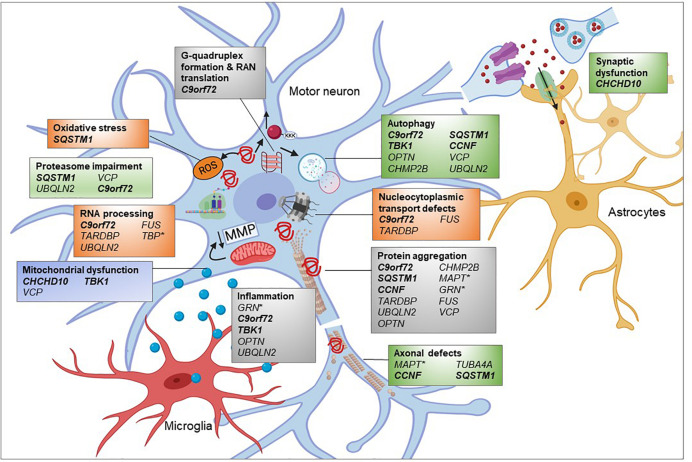
Pathogenic pathways associated with genetic variants of FTD. FTD is a complex disease attributed to multiple genetic mutations affecting several cellular pathways. Many of the proteins encoded by these genes are found to be aggregated into inclusions. Overload of these misfolded proteins might impair the proteasome and autophagy, affecting timely clearance of abnormal proteins. Microtubule dysfunction results in abnormal anterograde and retrograde axonal transport of vesicular cargoes and mitochondria and axonal degeneration. Decreased mitochondrial membrane potential (MMP) could damage mitochondrial function and subsequently promote exiting of TDP-43 from the nucleus contributing to cytoplasmic TDP-43 inclusions. RNA processing and nucleocytoplasmic transport defects impact upon many cellular pathways, depending on the regulatory RNAs and proteins being affected. Mutations in FTD-related genes such as progranulin (GRN) could promote inflammatory response by microglia which is toxic to neuronal health. Genes in bold and italic = ALS-FTD genes,^∗^ = genes only associated with FTD and the remainder are associated with ALS and FTD in differing proportions. Created using Biorender.com.

Whilst there are far fewer genes associated with only FTD, *GRN* (responsible for 5–20% of fFTD) and *MAPT* (responsible for 10–20% of fFTD) are also involved in similar pathways (Figure 3). The progranulin gene (*GRN*) encodes a secreted glycoprotein that is taken up by the cell and cleaved into multiple smaller granulins. The precise function of granulin is still to be determined, though it has been shown to be involved in multiple pathways including neuronal survival, neurite outgrowth, neuroinflammation, and autophagy (Olszewska et al., 2016). Mutations in *GRN*, leading to haploinsufficiency, are thought to cause FTD though lysosomal defects and reduced clearance of proteins (Ferrari et al., 2019). As with ALS, TDP-43 inclusions are also present, of the FTLD-TDP Type A form (Mackenzie et al., 2011). *MAPT*, encoding the microtubule associated protein tau, stabilizes microtubules through binding to tubulin. Mutations in *MAPT* disrupt this binding and lead to hyperphosphorylated tau aggregates. Recently, mutations in the TATA-box-binding gene (*TBP*), normally associated with spinocerebellar ataxia 17 (SCA17), were identified in a patient with FTD whose MRI showed cerebellar atrophy (Olszewska et al., 2019). The variant was found to co-segregate with disease. Thus, this new FTD gene, which encodes a transcription initiation factor can be categorized as an RNA processing gene.

The identification of a novel gene in a pathway that has not previously been associated with the genetics of ALS or FTD is particularly valuable for highlighting new disease biology and subsequently novel therapeutic targets. In this respect, the identification of mutations in *GLT8D1* and *DNAJC7* in fALS cases are notable discoveries. GLT8D1 is a glycosyltransferase with an enrichment of familial ALS-associated mutations proximal to the substrate binding site (Cooper-Knock et al., 2019). It was demonstrated that the mutations negatively impact enzyme activity suggesting a loss of function mechanism. Whilst the exact role of GLT8D1 remains to be discovered, however, glycosyltransferases are known to be involved in the synthesis of gangliosides which are signaling molecules important for motor neuron function (Harschnitz et al., 2014). As such, it is perhaps not surprising that glycosyltransferase dysfunction has already been associated with other neurodegenerative diseases such as Parkinson’s, Huntington’s and Alzheimer’s disease.

DNAJC7 encodes a heat shock protein (HSP40) which alongside HSP70 facilitates protein homeostasis, through folding new peptides and removing misfolded proteins. Rare protein truncating mutations were identified in *DNAJC7* in ALS cases and were absent from controls and subsequent screening identified further loss of function mutations as well as several rare missense mutations, predicted to be damaging (Farhan et al., 2020). In fibroblasts from a patient with a p.Arg156Ter mutation, protein levels were reduced, suggesting that these mutations may lead to protein aggregation, a characteristic feature of ALS. Further screening of *DNAJC7* and *GLT8D1* in additional cohorts of ALS, ALS-FTD, and FTD cases will establish the contribution of these genes and the roles of their proteins in disease pathogenesis.

## Conclusion

Amyotrophic lateral sclerosis and FTD have been described as forming a spectrum of disease, with converging mechanisms of neurodegeneration involving RNA processing, stress granules, protein aggregation and autophagy supporting this proposal (Ling et al., 2013; Deng et al., 2017; Nguyen et al., 2019; Baradaran-Heravi et al., 2020). However, it also clear that some genes, such as *MAPT*, are quite distinct and therefore it is argued that these should be kept separate (Hardy and Rogaeva, 2014). This distinction is also supported by the neuropathology, as whilst the majority of genetic (and sporadic) ALS cases have TDP-43-positive inclusions, along with *C9orf72* and *GRN*- FTD, *MAPT*- FTD does not, similar to *SOD1*-ALS. Thus, these distinctions are important to consider when pursuing diagnostic and prognostic biomarkers or therapeutic strategies.

The application of next generation sequencing, either in the form of targeted, whole exome or whole genome sequencing (WGS) has had a significant impact on the identification of genes associated with these diseases. However, it is also: (i) broadening the range of diseases that we see associated with variants in these genes (Table 2), (ii) broadening the range of genes that you would conventionally associate with ALS and FTD (Blauwendraat et al., 2018; Tripolszki et al., 2019), (iii) increasing the frequency of variants in known ALS and FTD genes within apparently sporadic cases, highlighting the variable penetrance of many of these proposed mutations (Tripolszki et al., 2019), (iv) identifying multiple variants in disease-associated genes within an individual (Cady et al., 2015), which will become increasingly important as personalized medicine based on your genetic mutation enters the clinic and finally (v) illustrating both the variability in frequencies of known genes across populations worldwide (Majounie et al., 2012; Wei et al., 2019), but also the inequality as the majority of these studies are undertaken in the northern hemisphere. It is hoped that WGS of large international cohorts of ALS and FTD such as Project MinE^2^ and GENFI^3^ will begin to fully understand the genetic contribution to disease and potentially answer why individuals with a particular variant go on to develop ALS, FTD or ALS-FTD.

**TABLE 2:** Clinical phenotypes also associated with ALS and FTD related genes.

ALS loci	Gene name	Alternative clinical phenotypes	Inheritance
FTDALS2	*CHCHD1*	SMA, Jokela type (SMAJ); Myopathy isolated mitochondrial, autosomal dominant (IMMD)	AD AD
FTDALS3	*SQSTM1*	Paget disease of bone 3 (PDB3); Myopathy, distal, with rimmed vacuoles (DMRV); Neurodegeneration with ataxia, dystonia and gaze palsy, childhood onset (NADGP)	AD AD AR
FTDALS4	*TBK1*	Encephalopathy, acute, infection-induced 8 (IIAE8) (susceptibility to)	AD
ALS1	*SOD1*	Spastic tetraplegia and axial hypotonia, progressive (STAHP)	AR
ALS2	*ALS2*	Primary lateral sclerosis, juvenile (PLSJ); Spastic paralysis, infantile onset ascending (IAHSP)	AR AR
ALS4	*SETX*	Spinocerebellar ataxia, with axonal neuropathy 2 (SCAN2)	AR
ALS5	*SPG11*	Spastic paraplegia 11 (SPG11); Charcot-Marie-Tooth disease, axonal, type 2X (CMT2X)	AR AR
ALS6	*FUS*	Tremor, hereditary essential 4 (ETM4)	AD
ALS8	*VAPB*	Spinal muscular atrophy (SMA), late onset, Finkel type (SMAFK)	AD
ALS11	*FIG4*	Charcot-Marie-Tooth disease, type 4J (CMT4J); Yunis-Varon syndrome Polymicrogyria, bilateral temporo-occitpital (BTOP)	AR AR AR
ALS12	*OPTN*	Glaucoma, primary open angle (POAG); Glaucoma, normal tension (susceptibility to)	AD
ALS13	*ATXN2*	Spinocerebellar ataxia 2 (SCA2); Parkinson’s disease, late onset (susceptibility to)	AD AD
ALS14	*VCP*	Charcot-Marie-Tooth disease, type 2Y (CMT2Y); Inclusion body myopathy with early onset Paget disease and frontotemporal dementia 1 (IBMPFD1)	AD AD
ALS16	*SIGMAR1*	SMA, distal, autosomal recessive 2 (DSMA2)	AR
ALS20	*hnRNPA1*	Inclusion body myopathy with early onset Paget disease and frontotemporal dementia 3 (IBMPFD3)	AD
ALS24	*NEK1*	Short-rib thoracic dysplasia 6, with or without polydactyly (SRTD6)	AR
ALS25	*KIF5A*	Spastic paraplegia 10 (SPG10); Myoclonus, intractable, neonatal (NEIMY)	AD AD

*Data obtained from Gene-Phenotype relationship data available on OMIM (http://www.ncbi.nlm.nih.gov/omim).*

## Author Contributions

RR, SH, KC, SS, JC-K, and JK wrote the sections of the manuscript. JK and RR designed and drew the figures. SH drafted Table 1. JK drafted Table 2. JK completed the review of all sections, final edits, and formatting. All authors contributed to the article and approved the submitted version.

## Conflict of Interest

The authors declare that the research was conducted in the absence of any commercial or financial relationships that could be construed as a potential conflict of interest. The handling editor declared a past co-authorship with two of the authors JK and JC-K.

## Footnotes

1 https://www.ncbi.nlm.nih.gov/omim

2 www.projectmine.com

3 http://genfi.org.uk/
